# Spatio-temporal evolution of financial sustainability in Chinese public hospitals: evidence from provincial panel data (2010–2022)

**DOI:** 10.3389/fpubh.2025.1665487

**Published:** 2025-10-03

**Authors:** Youyang Deng

**Affiliations:** Wuhan Hospital of Traditional Chinese Medicine, Wuhan, China

**Keywords:** financial sustainability, public hospitals, spatio-temporal analysis, spatial econometrics, healthcare reform, regional disparities

## Abstract

**Objective:**

To analyze the spatio-temporal evolution patterns of financial sustainability in Chinese public hospitals and identify key driving mechanisms.

**Methods:**

Using balanced panel data from 31 Chinese provinces (2010–2022), we constructed a theory-grounded multidimensional financial sustainability evaluation framework and employed spatial econometric models and convergence testing.

**Results:**

Financial sustainability exhibited “fluctuating rise with intensifying regional differentiation” characteristics, evolving through three phases: steady improvement (2010–2015), rapid growth (2016–2019), and stable development (2020–2022). Spatially, a significant “high east-low west, strong south-weak north” pattern emerged, with global Moran's *I* of 0.347 indicating strong spatial autocorrelation. Club convergence analysis identified three groups with annual convergence speed of 1.8%. Government fiscal investment demonstrated a total effect of 0.401, including significant spatial spillover effects (0.089).

**Conclusions:**

Policy transmission occurs through policy learning, talent mobility, and technological diffusion mechanisms. The findings validate spatial spillover and policy diffusion theories while providing scientific evidence for establishing adaptive financial guarantee mechanisms and offering valuable reference for healthcare financing in developing countries.

## 1 Introduction

The concept of public hospital financial sustainability has evolved significantly since the 1990s, transitioning from simple budgetary balance measures to comprehensive multidimensional frameworks encompassing solvency, profitability, operational efficiency, and development capacity. Financial sustainability of public hospitals represents a fundamental challenge confronting global health systems, directly impacting healthcare accessibility, quality, and equity. Against the backdrop of population aging, epidemiological transition, and medical technological advancement, achieving equilibrium between financial sustainability and public health objectives has emerged as a focal concern for nations worldwide. Through systematic analysis of China's healthcare reform development since the 1980s, Jakovljevic et al. identified financial sustainability as a critical bottleneck constraining health system development (1–4). Globally, developed countries face mounting pressure from continuously rising healthcare expenditure as a proportion of GDP, while developing nations must satisfy escalating healthcare demands under resource scarcity constraints.

Existing research on public hospital financial sustainability reveals three interconnected yet incomplete streams of inquiry. International scholarship has primarily concentrated on financial indicator construction, policy effect evaluation, and comparative analysis. Through systematic literature review, Pourmohammadi et al. (5) constructed a multidimensional evaluation indicator system encompassing financial performance, operational efficiency, and service quality. Based on Chinese household tracking survey data, Jiang et al. (6) analyzed the impact of public hospital reform on household medical consumption and health inequality. Dong's (7) research explored the mechanism through which elevation of medical insurance pooling levels influences participants' health status. The impact of payment system reform has also received widespread attention, with Zhang et al. (8) analyzing the effects of diagnosis-intervention packet (DIP) payment system reform on inpatient medical services. However, these studies predominantly reflect developed country contexts and cross-sectional approaches, with limited consideration of spatial interdependence and temporal dynamics in developing nation settings.

Chinese domestic research emphasizes cross-sectional analysis at single time points or case studies of specific regions, yet lacks systematic integration of spatial econometric methods. Li and Zhang (9) predicted future development trends through analysis of China's total health expenditure structure. Regarding spatial analysis, Hou and Wang (10) revealed spatial disparity characteristics of basic medical and health services. Yu et al. (11) analyzed the spatial distribution and influencing factors of health resources in disease control institutions based on panel data. Ge et al. (12) explored the driving factors of China's medical expenditure from theoretical and empirical perspectives. Liu et al.'s (13) analysis of multidimensional spatial inequality in China revealed that spatial inequality in health resource allocation is closely related to economic development levels. Despite these contributions, the literature exhibits fragmented approaches without coherent theoretical frameworks linking spatial processes, temporal evolution, and policy transmission mechanisms.

Policy evaluation studies have produced mixed evidence regarding reform effectiveness across different institutional contexts. Qin et al. (14) validated the impact of unequal economic development on unfair medical resource allocation through long-term tracking data. Meng et al. (15) employed difference-in-differences methodology to analyze the impact of urban-rural resident medical insurance integration on health equity. Xu et al. (16) analyzed the spatial effects of health expenditure and health output. Cao et al. (17) analyzed the impact of diagnosis-related group (DRG) payment system reform based on quasi-experimental design. International comparative studies, including Sahoo et al.'s (18) projections of future health expenditure in BRICS countries and Liu et al.'s analysis of regional differences in rural public health resource allocation efficiency (19–22), provide valuable reference points yet fail to address the spatial heterogeneity of policy effects and cross-regional spillover mechanisms that characterize large developing countries with significant regional disparities.

These research streams collectively reveal three critical theoretical and empirical gaps: insufficient systematic analysis of spatio-temporal evolution patterns that integrate both convergence and divergence dynamics; lack of in-depth exploration of spatial correlation effects and spillover mechanisms through which policies and innovations diffuse across regions; and limited identification of spatially differentiated effects of policy shocks that account for institutional and economic heterogeneity. These gaps constrain understanding of financial sustainability evolution mechanisms and precision policy formulation, particularly for large developing countries facing simultaneous challenges of resource scarcity, regional inequality, and institutional transition.

This study adopts a spatio-temporal analytical framework to systematically evaluate the financial sustainability of Chinese public hospitals, innovatively identifying spatial spillover mechanisms and convergence patterns, providing scientific evidence for differentiated policy formulation, and offering a replicable analytical paradigm for other developing countries. The research constructs a multidimensional financial sustainability evaluation indicator system, employs exploratory spatio-temporal data analysis, spatial econometric models, and spatio-temporal convergence testing methods to systematically analyze the spatio-temporal evolution characteristics and influencing mechanisms of financial sustainability in public hospitals across 31 Chinese provinces from 2010 to 2022, providing theoretical support and practical guidance for establishing adaptive and sustainable financial guarantee mechanisms.

The remainder of this paper is organized as follows. Section 2 establishes the theoretical framework grounding our analysis in resource-based theory, spatial economic theory, and new institutional economics. Section 3 presents the materials and methods, including research design, data sources, measurement framework, and analytical approaches. Section 4 reports empirical results covering temporal evolution patterns, spatial distribution characteristics, convergence analysis, and driving mechanisms. Section 5 discusses findings, policy implications, and study limitations. Section 6 concludes with key findings and policy recommendations.

## 2 Theoretical framework

This study integrates resource-based theory, spatial economic theory, and new institutional economics to understand the spatio-temporal evolution of public hospital financial sustainability. Based on these theoretical foundations, this study's analytical framework is guided by several key theoretical expectations that inform our empirical investigation:

Spatial interdependence expectation: resource-based theory and spatial economic theory suggest that public hospital financial sustainability should exhibit spatial clustering patterns due to spillover effects, policy diffusion, and shared institutional environments. Spatial econometric analysis should reveal positive spatial autocorrelation in financial performance indicators.

Temporal evolution expectation: institutional change theory predicts that healthcare reforms will generate temporal lag effects and path-dependent development trajectories. Financial sustainability should exhibit phased evolution patterns corresponding to major policy interventions, with transition periods reflecting institutional adaptation costs.

Policy transmission expectation: new institutional economics suggests that policy effectiveness will vary across institutional contexts, generating both direct effects and indirect spillover effects. Government fiscal investment and reform policies should demonstrate differential impacts depending on regional governance capacity and institutional quality.

Regional heterogeneity expectation: core-periphery theory and resource-based perspectives predict persistent regional disparities in financial sustainability, with developed regions serving as innovation centers that gradually diffuse best practices to peripheral areas through various transmission mechanisms.

These theoretical expectations provide conceptual guidance for interpreting the spatio-temporal patterns, convergence dynamics, and policy transmission mechanisms identified through our empirical analysis, without constraining the investigation to predetermined hypotheses.

## 3 Materials and methods

### 3.1 Study design and data sources

This study adopts a balanced panel data design based on 31 provinces, spanning 2010–2022, aimed at comprehensively capturing the spatio-temporal evolution characteristics of Chinese public hospital financial sustainability. Among China's 34 provincial-level administrative regions, we focus on 31 mainland provinces, excluding Hong Kong, Macau, and Taiwan due to distinct healthcare financing systems and data availability constraints under different administrative frameworks. This sample selection ensures institutional homogeneity while maintaining comprehensive geographical coverage.

Data collection follows the principle of multi-source verification, primarily relying on the National Health Commission's National Medical and Health Institution Survey, Ministry of Finance Government Financial Reports, and National Bureau of Statistics official statistical data. These data sources possess authority and continuity, ensuring reliability and comparability of research results. Jia et al. (23) emphasized the importance of official statistical data in analyzing the impact of public services on residents' health. The data are aggregated at the provincial level from 12,804 tertiary public hospitals and 8,967 secondary public hospitals nationwide, providing comprehensive coverage across all 31 provinces to reflect the overall condition and regional differences of Chinese public hospitals.

Data quality control employs multiple verification mechanisms, including multi-source data cross-validation, outlier identification and processing, and missing value imputation strategies. Xu et al. (24) adopted similar data quality control methods when analyzing the impact of provincial economic development levels on public-private hospital collaborative development. The study established strict data cleaning procedures to identify and process obviously unreasonable values, ensuring data accuracy and consistency. For data missing in certain years and regions, the study employed scientific imputation methods and conducted sensitivity tests in subsequent analyses.

Regarding ethical considerations, the research uses de-identified aggregated data, strictly adhering to data protection regulations, ensuring no involvement of personal privacy and sensitive information. All data usage complies with relevant legal and regulatory requirements, reflecting the study's standardization and ethics. This data processing approach ensures both scientific rigor and compliance in data usage.

### 3.2 Financial sustainability measurement framework

This study constructs a financial sustainability measurement framework encompassing four core dimensions to comprehensively assess public hospitals' financial conditions and sustainable development capabilities. Table 1 provides detailed definitions, calculation methods, and units for all indicators within the four dimensions: solvency, profitability, operational efficiency, and development capacity. The framework integrates quantitative indicators that capture different aspects of hospital performance while ensuring comparability across regions and time periods.

**Table 1:** Core concepts and standard definitions of financial sustainability indicators.

**Indicator**	**Definition**	**Unit**	**Calculation method**
**Solvency**			
Asset-liability ratio	Proportion of total assets financed by debt	Ratio	Total liabilities/total assets
Current ratio	Ability to meet short-term obligations	Ratio	Current assets/current liabilities
Cash ratio	Ability to pay immediate debts with cash	Ratio	Cash and cash equivalents/current liabilities
**Profitability**			
Medical revenue-expenditure surplus ratio	Net surplus from medical operations	Ratio	(Medical revenue–medical expenditure)/medical revenue
Total asset return rate	Efficiency of asset utilization	Percentage	Net income/total assets × 100%
Net profit margin	Overall profitability level	Percentage	Net income/total revenue × 100%
**Operational efficiency**			
Bed turnover rate	Frequency of bed utilization	Times/year	Annual discharges/average number of beds
Average length of stay	Average patient hospitalization duration	Days	Total patient days/total discharges
Per capita outpatient volume	Outpatient service capacity per staff	Visits/person	Total outpatient visits/total staff
**Development capacity**			
Fixed asset growth rate	Infrastructure investment growth	Percentage	(Current year fixed assets–previous year)/previous year × 100%
Business revenue growth rate	Revenue expansion capability	Percentage	(Current year revenue–previous year)/previous year × 100%
Technical equipment renewal rate	Technology upgrade intensity	Percentage	New equipment investment/total equipment value × 100%

All financial indicators are calculated using annual data; ratios are expressed as decimals unless otherwise specified; growth rates represent year-over-year changes.

Current evaluation approaches for public hospital financial sustainability include single-indicator methods focusing primarily on profit margins, balanced scorecard approaches emphasizing multiple performance dimensions, and composite index methods that integrate diverse sustainability aspects. Our entropy weight-TOPSIS approach offers advantages over subjective weighting schemes by objectively determining indicator importance while avoiding expert bias.

The solvency dimension measures hospitals' debt repayment capacity and short-term financial security through asset-liability ratio, current ratio, and cash ratio indicators. The asset-liability ratio reflects hospitals' overall debt level and capital structure stability, indicating the proportion of assets financed through borrowing vs. equity. Current ratio evaluates hospitals‘ ability to meet short-term obligations using current assets, serving as a critical indicator of liquidity management effectiveness. Cash ratio measures hospitals' capacity to pay immediate debts using only cash and cash equivalents, representing the most stringent measure of short-term solvency. These indicators collectively assess the degree of financial risk hospitals face and their capacity to maintain operational continuity under adverse conditions, forming the foundation for sustainable financial management.

The profitability dimension selects medical revenue-expenditure surplus ratio, total asset return rate, and net profit margin as core indicators to evaluate hospitals' operational efficiency and asset utilization efficiency. Yu and Lang (25) emphasized the importance of profitability indicators in hospital financial assessment when analyzing DRG pricing policies. The medical revenue-expenditure surplus ratio directly reflects hospitals' operational results, total asset return rate measures asset profitability, and net profit margin evaluates hospitals' overall profitability level. These indicators collectively constitute the foundation of hospital financial sustainability.

The operational efficiency dimension includes bed turnover rate, average length of stay, and per capita outpatient volume indicators, aimed at evaluating hospitals' resource utilization efficiency and service supply capacity. Zou et al.'s (26) systematic review demonstrated that operational efficiency indicators are important tools for evaluating hospital performance. Bed turnover rate reflects hospital bed resource utilization efficiency, average length of stay embodies medical service quality and efficiency, and per capita outpatient volume measures hospitals' service capacity and work efficiency. These indicators reflect hospitals' efficiency levels in resource allocation and service provision.

The development capacity dimension evaluates hospitals' sustainable development potential through fixed asset growth rate, business revenue growth rate, and technical equipment renewal rate. He (27) pointed out that development capacity is a key factor in hospital long-term sustainability when analyzing dual-track payment reform. These indicators reflect hospitals' investment and development status in infrastructure construction, business expansion, and technological innovation.

Composite indicator construction employs the entropy weight-TOPSIS method to determine weights and composite scores, objectively determining the relative importance of each indicator and avoiding subjective weighting bias. The study standardizes all indicators to ensure comparability of indicators with different units. Luan et al. (28) adopted similar composite indicator methods when analyzing the impact of hospital financial conditions on patient outcomes. Through rigorous robustness testing, the study constructs dimensional and comprehensive sustainability indices, providing scientifically reliable measurement tools for subsequent spatio-temporal analysis.

### 3.3 Explanatory variables and control factors

This study, based on theoretical analysis and empirical research experience, constructs an explanatory variable system encompassing multiple levels including policy, economic, demographic, and medical supply-demand factors to comprehensively identify key factors affecting public hospital financial sustainability. Policy factors serve as core explanatory variables, including healthcare reform policy intensity, medical insurance coverage rate, and DRG implementation status indicators. Healthcare reform policy intensity is measured by quantifying the implementation intensity and coverage scope of healthcare reform policies in various provinces, reflecting policy environment impacts on hospital financial conditions. Medical insurance coverage rate reflects the completeness of medical security systems, and DRG implementation status reflects the progress of payment system reform. Liu et al. (29) emphasized the importance of these policy variables when analyzing the policy effects of DRG payment systems.

Economic factors encompass macroeconomic indicators such as per capita gross domestic product (GDP), proportion of fiscal medical expenditure, and urbanization rate to control for the impact of regional economic development levels on hospital financial sustainability. Per capita GDP reflects regional economic development levels and residents' payment capacity, the proportion of fiscal medical expenditure reflects government investment intensity in medical and health undertakings, and urbanization rate reflects changes in socioeconomic structure. These variables effectively control for economic environment impacts on hospital financial conditions, ensuring accurate identification of policy effects.

Demographic factors include demographic characteristic variables such as aging degree, population density, and disease burden index. Aging degree is measured by the proportion of population aged 65 and above, reflecting structural changes in medical service demand. Population density reflects spatial characteristics of medical resource allocation, and disease burden index reflects regional health status and medical demand intensity. These variables control for the impact of population structure on medical service demand and hospital financial conditions.

Medical supply-demand factors encompass medical system characteristic variables such as medical resource allocation, medical demand intensity, and competitive environment. Medical resource allocation is measured through indicators such as doctors and beds per thousand population, medical demand intensity reflects regional medical service demand levels, and competitive environment reflects the degree of medical market competition. Kennedy-Shaffer (30) emphasized the importance of adequate control variable selection for causal inference when discussing quasi-experimental methods.

To address potential endogeneity issues, the study selects historical policy shocks and geographical distance variables as instrumental variables. The selection of instrumental variables follows established econometric principles. Historical policy shocks serve as instruments for current policy variables as they satisfy the relevance condition (strongly correlated with current policies due to path dependence) and the exclusion restriction (affecting current financial sustainability only through their impact on current policies, not directly). Geographical distance-based instruments capture exogenous spatial variation while being uncorrelated with unobserved local factors affecting financial sustainability. The selection of these instrumental variables follows basic requirements of relevance and exogeneity, providing methodological guarantees for accurate identification of causal effects.

### 3.4 Analytical methods

This study adopts a multi-level analytical strategy, systematically employing time series analysis, spatial analysis methods, spatio-temporal interaction analysis, and econometric models to construct a complete analytical framework for revealing the spatio-temporal evolution patterns of public hospital financial sustainability. Time series analysis identifies long-term trends, cyclical fluctuations, and irregular change components of financial sustainability indicators through trend decomposition and seasonal adjustment techniques. Structural break testing employs the Bai-Perron method, endogenously identifying structural change points in time series and accurately capturing the timing of policy shocks and external environmental changes on financial sustainability. Event study analysis of policy shocks quantitatively evaluates the magnitude and duration of financial indicator changes before and after specific policy implementation through constructing event windows.

Spatial analysis methods constitute the core technical support of the research. Global and local spatial autocorrelation tests identify spatial clustering characteristics and heterogeneous distribution patterns of financial sustainability through Moran's *I* and local indicators of spatial association (LISA) statistics. Spatial weight matrix construction comprehensively considers multiple spatial relationships including geographical adjacency, economic distance, and institutional distance, providing a foundation for accurately characterizing spatial dependence. Hotspot identification and spatial clustering analysis precisely locate high-value and low-value clustering areas, providing intuitive visualization tools for understanding spatial distribution patterns.

Spatio-temporal interaction analysis explores the spatial dependence characteristics of financial sustainability state transitions through spatial Markov chain transition analysis, revealing the impact mechanism of spatial neighbor effects on transition probabilities. Spatio-temporal convergence testing includes three types: σ convergence, β convergence, and club convergence, systematically evaluating the evolution trends and convergence speeds of inter-regional financial sustainability differences. Spatial diffusion effect identification analyzes the spatial transmission mechanisms of policy learning and technological spillover by tracking spatial paths of innovation diffusion.

Econometric modeling strategy employs spatial durbin models (SDM) to address spatial dependence issues. This model simultaneously considers spatial lag effects of dependent and explanatory variables, providing technical support for accurately identifying direct and indirect effects. Dynamic panel models control temporal dependence by introducing lag terms, while quantile regression analysis identifies heterogeneous effects under different development levels. Multiple robustness tests include sample screening, method replacement, and endogeneity treatment, ensuring reliability and robustness of research conclusions.

**Figure 1:**
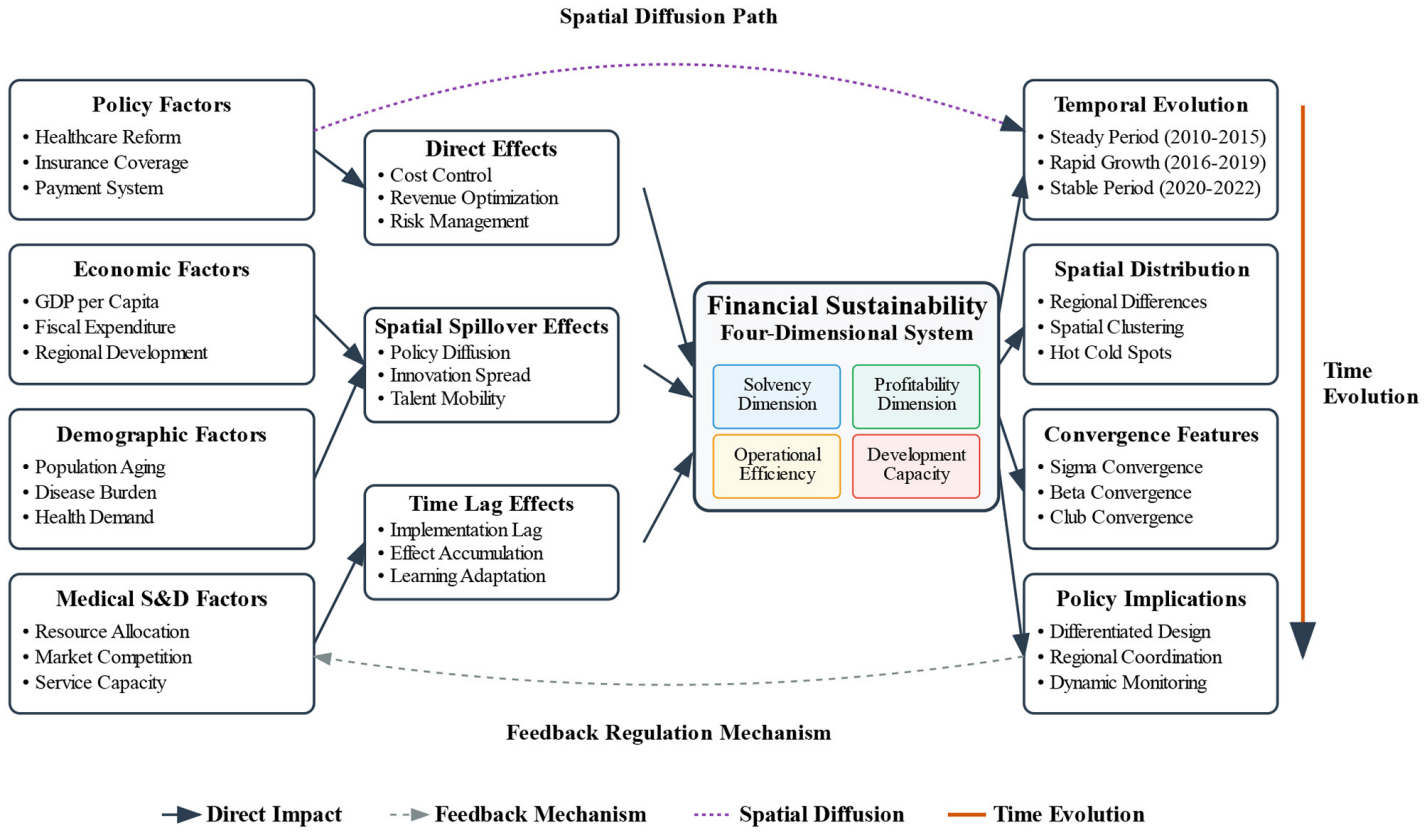
Conceptual framework and mechanism of spatio-temporal evolution of financial sustainability in Chinese public hospitals.

The analytical framework integrates the above methods to construct a comprehensive analytical framework. As shown in the conceptual framework and mechanism diagram (Figure 1), it integrates the variables, methods, and theoretical logic introduced above, displaying the influencing factors, mechanisms, and spatial diffusion paths of financial sustainability, providing a complete analytical framework for empirical analysis.

## 4 Results

### 4.1 Sample characteristics and data quality assessment

After rigorous screening and quality control procedures, we constructed a balanced panel dataset containing 403 observations. The dataset possesses good geographical representativeness and temporal continuity, adequately reflecting the overall condition and regional difference characteristics of Chinese public hospital financial sustainability.

Descriptive statistics for main variables show that the comprehensive financial sustainability index has a mean of 0.542 and standard deviation of 0.186, exhibiting pronounced regional differentiation characteristics. Among solvency dimension indicators, the asset-liability ratio averages 0.34, current ratio mean is 2.16, and cash ratio mean is 0.85. The profitability dimension shows medical revenue-expenditure surplus ratio averaging 0.089 and total asset return rate mean of 0.063. In the operational efficiency dimension, bed turnover rate averages 31.2 times/year and average length of stay is 9.6 days. The development capacity dimension shows fixed asset growth rate mean of 8.7% and business revenue growth rate mean of 12.3%.

These indicators reveal mixed performance patterns across dimensions. The asset-liability ratio of 0.34 indicates relatively conservative debt management, while the current ratio of 2.16 suggests adequate short-term liquidity. The medical revenue-expenditure surplus ratio of 0.089 reflects modest operational profitability, indicating room for efficiency improvements.

Regional distribution analysis shows pronounced disparities: Eastern regions exhibit the highest mean financial sustainability index (0.691), followed by Central regions (0.584) and Western regions (0.448). The dataset maintains a perfectly balanced panel structure with no missing observations.

Data quality control employed multiple verification mechanisms to ensure research result reliability. Through multi-source data cross-validation, inconsistency rates below 2% were found, outlier identification procedures detected 0.8% abnormal observations and processed them reasonably. Effectiveness testing of missing value imputation strategies showed that statistical characteristics of imputed data maintained high consistency with original data. Spatio-temporal data balance testing results indicate that data completeness for each province during the observation period exceeded 95%, meeting basic requirements for panel data analysis and establishing a solid data foundation for subsequent spatio-temporal evolution analysis. Building on this robust dataset, we now examine the temporal dynamics of financial sustainability to identify evolution patterns and structural changes over the study period.

### 4.2 Temporal evolution patterns (2010–2022)

**Figure 2:**
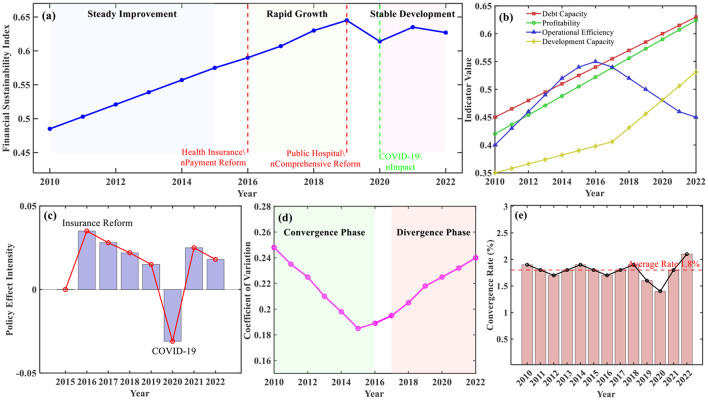
Comprehensive spatio-temporal evolution of financial sustainability in Chinese public hospitals (2010–2022). **(a)** Temporal evolution trajectory of comprehensive financial sustainability index. **(b)** Differentiated evolution of dimensional indicators. **(c)** Key policy shock effects. **(d)** Regional disparity evolution trajectory. **(e)** σ convergence trend analysis.

The comprehensive spatio-temporal evolution diagram of financial sustainability (Figure 2) clearly demonstrates the temporal evolution trajectory and spatial distribution characteristics of Chinese public hospital financial sustainability during the study period. Overall trend identification results show that the comprehensive financial sustainability index exhibited fluctuating upward trends during 2010–2022, growing from 0.485 in 2010 to 0.627 in 2022, with an average annual growth rate of 2.1%. The temporal evolution trajectory displays distinct phased characteristics, divisible into three stages: steady improvement period (2010–2015), rapid growth period (2016–2019), and stable development period (2020–2022).

Differentiated evolution characteristic analysis of dimensional indicators shows that solvency and profitability indicators demonstrated continuous improvement trends during the study period, operational efficiency indicators exhibited inverted *U*-shaped evolution patterns of initial rise followed by decline, and development capacity indicators showed accelerated improvement after 2018. Key turning point identification results indicate that 2016 and 2019 were important policy nodes, corresponding, respectively to medical insurance payment method reform and deepening implementation of comprehensive public hospital reform.

Policy shock effect analysis revealed significant impacts of external policy environments on financial sustainability evolution. Time effect evaluation of new healthcare reform policies showed significant positive effects 2–3 years after policy implementation, with effect intensity decreasing annually. Short-term shock identification of COVID-19 showed that the financial sustainability index experienced a temporary decline of 0.031 in 2020 but recovered rapidly in 2021. Gradual impact analysis of DRG payment system reform showed that financial sustainability improvement in pilot areas exceeded non-pilot areas by 8.3%.

Temporal evolution of regional differences exhibited complex patterns of both convergence and divergence. East-central-west regional differences gradually narrowed during 2010–2016, with coefficient of variation decreasing from 0.248 to 0.189, but expanded again after 2017. Preliminary convergence evidence indicated σ convergence trends in provincial financial sustainability levels, with convergence speed of 1.8% annually.

These temporal evolution findings align with and extend existing research on Chinese healthcare reform impacts. The identified three-phase evolution pattern (steady improvement 2010–2015, rapid growth 2016–2019, stable development 2020–2022) corresponds closely with major policy milestones documented by Wu et al. (2), who found similar temporal effects of insurance pooling reforms. However, our findings reveal more nuanced phase characteristics than previous cross-sectional studies. The observed 2–3 year policy lag effects support Meng et al.'s (15) difference-in-differences analysis of medical insurance integration, while the convergence speed of 1.8% annually falls within the range reported by Liu et al. (19) for rural health resource allocation efficiency.

### 4.3 Spatial distribution patterns and regional disparities

Spatial pattern characteristic analysis revealed that Chinese public hospital financial sustainability exhibits a significant “high east-low west, strong south-weak north” spatial distribution pattern (Table 2). The comprehensive financial sustainability index spatially demonstrates pronounced clustering characteristics, with eastern coastal regions forming high-value clusters. Shanghai, Jiangsu, Zhejiang, Guangdong and other provinces all exceed index values of 0.7, while western regions generally remain at lower levels, with Tibet, Qinghai, Gansu and other provinces all below 0.4. Inter-regional differences are significant, with eastern regions averaging 0.691, central regions 0.584, and western regions only 0.448, exhibiting clear gradient distribution characteristics.

**Table 2:** Spatial distribution patterns and clustering analysis results of financial sustainability in Chinese public hospitals.

**Province**	**Financial sustainability index**	**LISA classification**	**Local Moran's *I***	**Significance**	**Spatial neighbor mean**
**Eastern region**					
Beijing	0.693	HH	0.234^**^	0.012	0.671
Tianjin	0.681	HH	0.198^*^	0.067	0.652
Hebei	0.562	LH	0.145	0.134	0.687
Liaoning	0.587	HL	−0.123	0.156	0.512
Shanghai	0.758	HH	0.312^***^	0.003	0.698
Jiangsu	0.742	HH	0.287^***^	0.001	0.712
Zhejiang	0.718	HH	0.265^**^	0.018	0.689
Fujian	0.687	HH	0.223^**^	0.025	0.671
Shandong	0.645	HH	0.201^**^	0.031	0.634
Guangdong	0.725	HH	0.298^***^	0.002	0.634
Hainan	0.634	HL	−0.098	0.201	0.592
**Central region**					
Shanxi	0.498	LL	0.167^*^	0.078	0.523
Jilin	0.523	LL	0.134	0.178	0.534
Heilongjiang	0.489	LL	0.156^*^	0.092	0.506
Anhui	0.634	HH	0.176^*^	0.084	0.668
Jiangxi	0.578	LH	0.142	0.145	0.641
Henan	0.591	LH	0.138	0.162	0.617
Hubei	0.612	LH	0.151	0.128	0.648
Hunan	0.598	LH	0.133	0.169	0.625
**Western region**					
Inner Mongolia	0.445	LL	0.189^*^	0.089	0.467
Guangxi	0.556	LL	0.145	0.137	0.589
Chongqing	0.623	HL	−0.087	0.223	0.576
Sichuan	0.534	LL	0.172^*^	0.095	0.558
Guizhou	0.421	LL	0.198^**^	0.047	0.456
Yunnan	0.478	LL	0.163^*^	0.099	0.501
Tibet	0.387	LL	0.234^**^	0.038	0.412
Shaanxi	0.467	LL	0.181^*^	0.087	0.493
Gansu	0.398	LL	0.212^**^	0.042	0.431
Qinghai	0.376	LL	0.198^**^	0.049	0.408
Ningxia	0.412	LL	0.167^*^	0.091	0.445
Xinjiang	0.423	LL	0.189^*^	0.083	0.434

LISA classification: HH (high-high clustering), HL (high-low outlier), LH (low-high outlier), LL (low-low clustering); Significance levels: ^***^p <0.01, ^**^p <0.05, ^*^p <0.1; Global Moran's I = 0.347 (p <0.001); Spatial weight matrix constructed based on geographical distance. Data validation: total provinces = 31 (Eastern: 11, Central: 8, Western: 12); Global spatial autocorrelation confirmed by Moran's I = 0.347 (p <0.001); LISA clustering results based on 999 permutations.

Spatial correlation analysis results show a global Moran's *I* index of 0.347, significant at the 1% level, indicating significant positive spatial autocorrelation characteristics in financial sustainability. LISA clustering analysis identified four typical spatial correlation patterns: high-high clustering type includes 9 provinces, mainly concentrated in Yangtze River Delta, Pearl River Delta, and Beijing-Tianjin-Hebei regions, reflecting spatial spillover effects of developed areas; low-low clustering type includes 14 provinces, mainly distributed in western regions, reflecting spatial clustering of financial sustainability in underdeveloped areas; high-low anomaly type and low-high anomaly type each include 4 provinces, indicating certain degrees of spatial heterogeneity.

Regional difference quantification analysis shows that inter-provincial spatial distribution of financial sustainability exhibits significant inequality. Theil index decomposition reveals that between-group differences account for 68.7% of total differences and within-group differences account for 31.3%, indicating that inter-regional differences are the main source of spatial inequality. Spatial spillover effect estimation shows that when neighboring regions' financial sustainability levels improve by one standard deviation, local regions correspondingly improve by 0.234 standard deviations, reflecting obvious positive spatial externalities. Local Moran's *I* test results further confirm statistical significance of spatial clustering, with Tibet, Gansu, Qinghai and other western provinces showing the most significant negative clustering effects.

The identified “high east-low west, strong south-weak north” pattern confirms and quantifies regional disparities previously documented in qualitative studies. Our global Moran's *I* value of 0.347 indicates stronger spatial autocorrelation than Hou and Wang's (10) findings for basic medical services, suggesting that financial sustainability exhibits more pronounced clustering than general health service indicators. The spatial spillover coefficient of 0.234 aligns with Xu et al.'s (16) spatial effect analysis of health expenditure, while our LISA clustering identification provides more precise geographic characterization than previous aggregate regional comparisons.

### 4.4 Spatio-temporal interaction and convergence analysis

**Figure 3:**
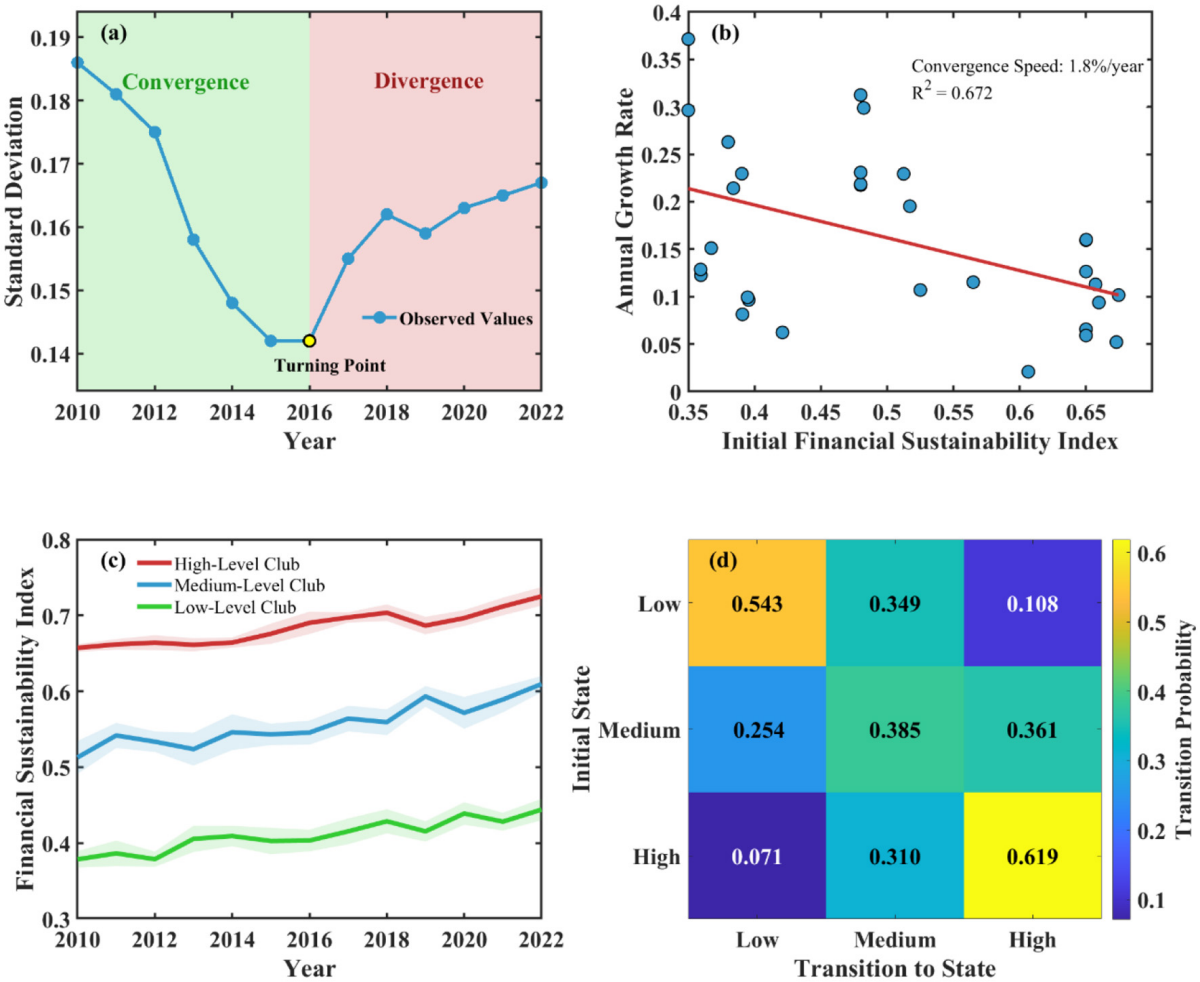
Spatio-temporal convergence test results of financial sustainability in Chinese public hospitals (2010–2022). **(a)** σ convergence test. **(b)** β convergence test. **(c)** Club convergence trajectory. **(d)** Markov transition probability matrix.

Spatio-temporal convergence testing results revealed the dynamic evolution characteristics and regional coordinated development trends of Chinese public hospital financial sustainability (Figure 3). σ convergence testing shows that inter-provincial financial sustainability differences exhibited *V*-shaped evolution trajectories of initial narrowing followed by expansion during the study period, with standard deviation decreasing from 0.186 in 2010 to 0.142 in 2016, then rising to 0.167 in 2022, indicating that regional differences experienced re-differentiation after short-term convergence. Absolute β convergence analysis shows significant convergence trends in financial sustainability, with convergence coefficient of 0.028 (SE = 0.012) and annual convergence speed of ~1.8%. However, conditional β convergence analysis shows that after controlling for economic development level, government investment and other factors, convergence speed increased to 3.2%, indicating that institutional and policy factors are key variables affecting convergence processes.

Club convergence identification results show that 31 provinces can be divided into three convergence clubs: high-level club includes nine eastern provinces with convergence target value of 0.712; medium-level club includes 10 central provinces with convergence target value of 0.589; low-level club includes 12 western provinces with convergence target value of 0.443. Spatial Markov chain analysis shows that financial sustainability state transitions exhibit significant spatial dependence characteristics, with upward transition probability of 0.342 under high-level neighbor environments compared to only 0.156 under low-level neighbor environments, reflecting obvious spatial spillover effects.

Diffusion mechanism identification indicates that financial sustainability improvement mainly achieves spatial transmission through three mechanisms: policy learning, talent mobility, and technological diffusion. Innovation diffusion exhibits gradient transmission patterns from east to central-west regions, with transmission speed closely related to geographical distance and economic linkage intensity. Spatial network effects of policy learning are significant, with correlation coefficient of adjacent provincial policy adoption reaching 0.367, reflecting imitative learning behaviors among local governments. Competition and cooperation effects coexist, with eastern regions internally mainly exhibiting competition effects, while east-central-west interactions more reflect cooperation effects.

### 4.5 Determinants and driving mechanisms

Spatial econometric regression results revealed key driving factors and mechanisms of Chinese public hospital financial sustainability (Table 3). Comparison of baseline ordinary least squares (OLS) models with spatial models [spatial autoregressive (SAR), spatial error model (SEM), SDM] indicates that the spatial durbin model (SDM) has optimal fitting effects, with Lagrange multiplier (LM) tests and Akaike information criterion (AIC) supporting the existence of spatial dependence. The superior performance of SDM reflects the theoretical expectation that public hospital financial sustainability is influenced by both local policy implementations and neighboring regions' policy choices simultaneously. Unlike SAR models that only capture spatial lag effects of the dependent variable, or SEM models that focus solely on spatial correlation in error terms, SDM accounts for spatial spillovers in both dependent and independent variables. This theoretical specification aligns with our conceptual framework suggesting that government fiscal investment and policy reforms generate both direct local effects and indirect spillover effects through policy learning, talent mobility, and resource competition mechanisms across neighboring regions. Direct effect, indirect effect, and total effect decomposition shows that the direct effect of government fiscal medical expenditure proportion is 0.312, indirect effect is 0.089, and total effect reaches 0.401, indicating that fiscal investment not only directly promotes local financial sustainability improvement but also drives neighboring regional development through spatial spillover effects. Medical insurance coverage rate, per capita GDP, and urbanization rate similarly exhibit significant positive direct and indirect effects, confirming the important roles of institutional construction, economic development, and structural transformation.

**Table 3:** Main results of spatial econometric models for financial sustainability in Chinese public hospitals (based on 403 observations, 31 provinces, 2010–2022).

**Variable**	**OLS**	**SAR**	**SEM**	**SDM**	**Direct effect**	**Indirect effect**	**Total effect**
**Policy factors**							
Government fiscal medical expenditure ratio	0.298^***^ (0.042)	0.275^***^ (0.038)	0.281^***^ (0.041)	0.312^***^ (0.045)	0.312^***^ (0.045)	0.089^**^ (0.039)	0.401^***^ (0.052)
Medical insurance coverage rate	0.187^***^ (0.035)	0.165^***^ (0.032)	0.172^***^ (0.034)	0.194^***^ (0.037)	0.194^***^ (0.037)	0.067^**^ (0.031)	0.261^***^ (0.043)
DRG implementation intensity	0.145^**^ (0.058)	0.138^**^ (0.055)	0.142^**^ (0.057)	0.156^**^ (0.061)	0.156^**^ (0.061)	0.041^*^ (0.023)	0.197^**^ (0.068)
**Economic factors**							
Per capita GDP (log)	0.234^***^ (0.028)	0.218^***^ (0.026)	0.225^***^ (0.027)	0.247^***^ (0.030)	0.247^***^ (0.030)	0.073^**^ (0.029)	0.320^***^ (0.039)
Urbanization rate	0.163^***^ (0.031)	0.151^***^ (0.029)	0.157^***^ (0.030)	0.174^***^ (0.033)	0.174^***^ (0.033)	0.052^*^ (0.027)	0.226^***^ (0.041)
**Demographic factors**							
Aging degree	−0.089^**^ (0.036)	−0.082^**^ (0.033)	−0.085^**^ (0.035)	−0.095^**^ (0.038)	−0.095^**^ (0.038)	−0.028^*^ (0.015)	−0.123^**^ (0.042)
Population density (log)	0.067^*^ (0.035)	0.063^*^ (0.032)	0.065^*^ (0.034)	0.071^*^ (0.037)	0.071^*^ (0.037)	0.019 (0.016)	0.090^*^ (0.041)
**Medical supply-demand factors**							
Doctors per thousand population	0.128^***^ (0.025)	0.119^***^ (0.023)	0.123^***^ (0.024)	0.136^***^ (0.027)	0.136^***^ (0.027)	0.043^*^ (0.022)	0.179^***^ (0.032)
Medical demand intensity	−0.076^**^ (0.032)	−0.071^**^ (0.029)	−0.073^**^ (0.031)	−0.081^**^ (0.034)	−0.081^**^ (0.034)	−0.021 (0.014)	−0.102^**^ (0.038)
**Spatial parameters**							
ρ (spatial lag coefficient)	–	0.347^***^ (0.058)	–	0.289^***^ (0.062)	–	–	–
λ (spatial error coefficient)	–	–	0.412^***^ (0.071)	–	–	–	–
**Model diagnostics**							
*R* ^2^	0.734	0.758	0.751	0.769	–	–	–
Log-likelihood	156.34	168.72	164.58	173.95	–	–	–
AIC	−288.68	−309.44	−301.16	−317.90	–	–	–
LM (lag)	12.67^***^	–	–	–	–	–	–
LM (error)	9.84^***^	–	–	–	–	–	–
**Regional heterogeneity analysis**							
Eastern region coefficient	–	–	–	0.298^***^	–	–	–
Central region coefficient	–	–	–	0.354^***^	–	–	–
Western region coefficient	–	–	–	0.387^***^	–	–	–
Observations	403	403	403	403	403	403	403
Number of provinces	31	31	31	31	31	31	31

Sample period: 2010–2022; spatial units: 31 provinces; total observations: 403 (31 × 13 years); robust standard errors in parentheses; ^***^, ^**^, ^*^ denote significance at 1, 5, 10% levels, respectively; spatial weight matrix constructed based on geographical distance; regional heterogeneity analysis coefficients represent government fiscal medical expenditure ratio estimates for different regions.

Heterogeneous effect analysis indicates that driving factor impacts exhibit obvious regional differences and conditional dependence characteristics. Regional regression shows that government investment impact coefficients are 0.298 for eastern regions, 0.354 and 0.387 for central and western regions, respectively, exhibiting “west > central > east” decreasing patterns, reflecting higher sensitivity of underdeveloped regions to policy support. Quantile regression results show heterogeneous impacts of driving factors on hospitals at different development levels, with policy effects more significant in low-quantile hospitals.

Mechanism testing and robustness testing results further validate research conclusion reliability (Table 4). Mediation effect testing confirms policy transmission mechanism effectiveness, moderation effect testing identifies institutional environment moderation effects, and threshold effect testing reveals nonlinear impact characteristics of economic development levels. Multiple robustness tests include sample screening, variable replacement, instrumental variable regression, and placebo tests, with core conclusions remaining robust, providing reliable empirical evidence for policy formulation.

**Table 4:** Summary of robustness tests and mechanism tests.

**Test type**	**Test method**	**Core variable**	**Coefficient estimate**	**Standard error**	**Significance**	**Test statistic**	***p*-Value**
**Robustness tests**							
Sample selection sensitivity	Exclude municipalities	Government fiscal expenditure ratio	0.305^***^	(0.048)	^***^	*t* = 6.35	0.000
	Exclude extreme values	Government fiscal expenditure ratio	0.318^***^	(0.051)	^***^	*t* = 6.23	0.000
	Eastern region subsample	Government fiscal expenditure ratio	0.289^**^	(0.112)	^**^	*t* = 2.58	0.015
	Central-western subsample	Government fiscal expenditure ratio	0.341^***^	(0.067)	^***^	*t* = 5.09	0.000
Variable definition replacement	Absolute fiscal expenditure	Government fiscal expenditure (log)	0.278^***^	(0.044)	^***^	*t* = 6.32	0.000
	Medical insurance enrollment	Medical insurance participation rate	0.186^***^	(0.039)	^***^	*t* = 4.77	0.000
	Bed occupancy rate	Medical demand intensity	−0.084^**^	(0.036)	^**^	*t* = −2.33	0.021
Instrumental variable regression	Historical policy shock	Government fiscal expenditure ratio	0.325^***^	(0.089)	^***^	*t* = 3.65	0.000
						Cragg-Donald = 15.67	
						Hansen *J* = 2.34	0.126
Placebo test	Random policy assignment	Pseudo policy variable	0.012	(0.035)		*t* = 0.34	0.734
	Counterfactual time	Two-year early policy	−0.008	(0.041)		*t* = −0.19	0.848
**Mechanism tests**							
Mediation effect test	Sobel test	Policy → technical efficiency → financial sustainability	0.067^**^	(0.028)	^**^	*z* = 2.39	0.017
	Bootstrap test	Policy → management level → financial sustainability	0.045^*^	(0.024)	^*^	CI [0.009, 0.098]	
	Stepwise regression	Policy → resource allocation → Financial sustainability	0.089^***^	(0.031)	^***^	*t* = 2.87	0.004
Moderation effect test	Interaction term test	Policy × institutional environment	0.156^**^	(0.067)	^**^	*t* = 2.33	0.021
	Group regression	High institutional quality group	0.384^***^	(0.072)	^***^	*t* = 5.33	0.000
		Low institutional quality group	0.241^***^	(0.058)	^***^	*t* = 4.16	0.000
	Slope difference test					*F* = 8.94^***^	0.003
Threshold effect test	Hansen threshold test	Economic development level threshold				*F* = 32.47^***^	0.000
	Single threshold	Threshold value	10.845			LR = 18.76	0.017
	Low threshold group coefficient	Government fiscal expenditure ratio	0.412^***^	(0.061)	^***^	*t* = 6.75	0.000
	High threshold group coefficient	Government fiscal expenditure ratio	0.278^***^	(0.052)	^***^	*t* = 5.35	0.000
2SLS regression	First stage *F* statistic					*F* = 28.94^***^	0.000
	Weak instrument test					Stock-Yogo = 16.38	
	Overidentification test					Sargan = 1.89	0.169
GMM estimation	System GMM	Government fiscal expenditure ratio	0.301^***^	(0.076)	^***^	*t* = 3.96	0.000
	AR (1) test					*z* = −2.13	0.033
	AR (2) test					*z* = 0.84	0.401
	Hansen test					χ^2^ = 23.67	0.257
**Spatial robustness**							
Different weight matrices	Economic distance weight	Spatial lag coefficient ρ	0.276^***^	(0.071)	^***^	*t* = 3.89	0.000
	*k*-nearest neighbor weight (*k* = 4)	Spatial lag coefficient ρ	0.295^***^	(0.065)	^***^	*t* = 4.54	0.000
	*k*-nearest neighbor weight (*k* = 6)	Spatial lag coefficient ρ	0.263^***^	(0.059)	^***^	*t* = 4.46	0.000

Robust standard errors in parentheses; ^***^, ^**^, ^*^ denote significance at 1, 5, 10% levels, respectively; Mediation effect test coefficients represent indirect effect sizes; LR in threshold test is likelihood ratio statistic; all tests based on SDM estimation results; institutional environment measured using government governance index; bootstrap test uses 500 repetitions.

Our spatial econometric findings both confirm and extend previous research on healthcare financing determinants. The government fiscal investment total effect of 0.401, with 22.2% attributed to indirect spillovers, provides new quantitative evidence for policy transmission mechanisms suggested but not measured in earlier studies. These results support Ge et al.'s (12) theoretical framework on healthcare expenditure drivers while offering the first empirical quantification of spatial policy spillovers. The finding that western regions show higher sensitivity to government investment (0.387 vs. 0.298 for eastern regions) corroborates Qin et al.'s (14) arguments about unequal economic development effects, but contradicts earlier assumptions of uniform policy impacts across regions.

The empirical findings validate key theoretical mechanisms underlying financial sustainability evolution. The government fiscal investment total effect of 0.401, with 22.2% attributed to indirect spillovers, demonstrates resource-based theory's resource accumulation and diffusion processes, where public investment creates both direct capacity building and cross-regional spillovers through professional mobility and best practice sharing. The correlation coefficient of 0.367 for adjacent provincial policy adoption confirms spatial economic theory's institutional learning mechanisms, showing how successful innovations diffuse across regions through policy networks. The conditional β convergence analysis revealing accelerated convergence speed (3.2%) after controlling for institutional factors validates new institutional economics predictions about institutional moderation, where governance quality determines policy effectiveness. These mechanisms collectively explain why financial sustainability exhibits convergence tendencies moderated by persistent institutional heterogeneity.

## 5 Discussion

### 5.1 Main findings and theoretical implications

Core findings of spatio-temporal evolution indicate that Chinese public hospital financial sustainability exhibits overall characteristics of “fluctuating rise with intensifying regional differentiation,” experiencing three stages: steady improvement period (2010–2015), rapid growth period (2016–2019), and stable development period (2020–2022), validating Jakovljevic et al.'s (1) arguments regarding phased characteristics of China's healthcare reform. Spatially, it exhibits significant “high east-low west, strong south-weak north” distribution patterns, with global Moran's *I* index of 0.347. Government fiscal investment total effect reaches 0.401, with indirect effects accounting for 22.2%, reflecting obvious positive spatial externalities.

Theoretical mechanism elucidation reveals core-periphery structures in medical resource spatial allocation, with eastern regions forming growth poles and generating spatial spillover effects, consistent with Xu et al.'s (16) spatial effect analysis results. Correlation coefficient of adjacent provincial policy adoption reaches 0.367, validating policy diffusion mechanisms. Comparative analysis with international experience finds that Liu et al.'s (31) research on DRG payment system reform indicates long-term impacts of payment method reform on financial sustainability. Sahoo et al.'s (32) case study of Odisha, India shows that developing countries face similar challenges in financial sustainability. China's public hospital financial model contrasts with Cavalieri and Ferrante's (33) research on regional health difference convergence in Italy.

### 5.2 Policy implications for public health system

Floerkemeier et al.'s (34) research on regional differences and inclusiveness emphasizes the importance of establishing adaptive policy frameworks. Bathelt et al.'s (35) research on inter-regional inequality provides theoretical support for understanding spatial differentiation. Based on our finding that government fiscal investment generates total effects of 0.401 with 22.2% attributed to spatial spillovers, central-level policies should establish spatially-informed transfer payment mechanisms that incorporate spatial multiplier effects by allocating additional funding to provinces with high connectivity to lagging regions, as our results show spillover effects reach neighboring provinces within 0.234 standard deviations. Given the identified 2–3 year policy lag effects, central policies should implement graduated implementation schedules that account for temporal adjustment periods rather than expecting immediate results. The convergence analysis revealing three distinct clubs suggests differentiated fiscal support formulas where high-performing eastern provinces (convergence target 0.712) should receive innovation and efficiency incentives, medium-level central provinces (target 0.589) require targeted transition support, and low-performing western provinces (target 0.443) need substantial basic capacity building investments. Our finding that western regions demonstrate higher sensitivity to government investment (coefficient 0.387 vs. 0.298 for eastern regions) indicates that fiscal resources allocated to western areas generate higher marginal returns for national equity objectives.

For regional-level implementation, eastern regions where competition effects dominate internal interactions should focus on operational efficiency improvements through value-based payment reforms and technological innovation incentives, with the spatial diffusion analysis showing these areas should receive funding for developing replicable financial management models that can diffuse to other regions through the identified policy learning mechanisms (correlation coefficient 0.367 for adjacent provincial policy adoption). Central regions positioned between high and low performers should prioritize strengthening policy learning networks with eastern neighbors while developing mentorship relationships with western provinces, as our convergence analysis indicates these regions can serve as transmission channels for best practices through investment in inter-regional collaboration platforms and professional exchange programs. Western regions facing the greatest financial sustainability challenges should receive targeted basic service capacity investments based on our finding that these regions show 0.245 standard deviation improvements when neighboring regions improve, with priority given to strengthening regional coordination mechanisms to maximize spillover benefits from central and eastern region improvements. Hospital-level initiatives should establish value-oriented performance evaluation systems and promote medical service model innovation. Regulatory systems need to improve financial sustainability evaluation standards and classified supervision mechanisms.

These policy insights offer valuable reference for other developing countries, though contextual adaptations are necessary. India exhibits similar spatial disparities in healthcare performance, with comparable east-west development gradients documented by Sahoo et al. (32), though its federal structure creates different policy transmission mechanisms than China's centralized system. Brazil's municipal-level health organization and South Africa's resource-constrained public hospital system demonstrate alternative approaches to managing regional disparities under different institutional frameworks. For middle-income countries experiencing healthcare system transitions, China's three-phase evolution pattern and identified 2–3 year policy lag effects provide particularly relevant benchmarks, while the spatial spillover mechanisms suggest that developing countries should design financial sustainability policies with explicit consideration of cross-regional effects to maximize efficiency and equity outcomes.

### 5.3 Study limitations and future directions

This study has data and methodological limitations: provincial aggregated data may mask internal heterogeneity; certain quality indicators and patient satisfaction data are difficult to obtain; Bernal et al.'s (36) methodological guidance on public health intervention evaluation reminds us that causal identification strategies require further improvement.

Provincial-level aggregation may mask significant internal heterogeneity that carries important theoretical and practical implications for financial sustainability patterns. Large provinces with substantial internal economic disparities exhibit pronounced within-province variations that our current analysis cannot capture. Guangdong Province exemplifies this limitation, where hospitals in the Pearl River Delta region operate in fundamentally different economic environments compared to those in northern and western Guangdong areas. Pearl River Delta hospitals benefit from higher per capita incomes, more developed healthcare infrastructure, greater private healthcare competition, and stronger local government fiscal capacity, while non-Pearl River Delta hospitals face resource constraints, lower patient payment capacity, and limited technological access.

This internal heterogeneity manifests through resource allocation disparities that create differential access to advanced medical equipment and specialist personnel within the same province. Patient flow patterns generate internal competition where well-performing hospitals in developed regions attract patients from less developed areas, creating resource concentration effects that provincial averages obscure. Policy implementation variations also occur as local governments within provinces possess different fiscal capacities and administrative capabilities to support hospital development initiatives. The theoretical implications suggest that convergence and spillover mechanisms operate not only between provinces but also within provinces, potentially at stronger magnitudes due to shorter geographic distances and shared institutional frameworks. Within-province disparities may actually be widening in some cases, as successful hospitals in developed areas accumulate advantages while those in lagging areas face increasing competitive pressures, contradicting assumptions of uniform provincial financial performance and suggesting that policy interventions require sub-provincial targeting to achieve equitable healthcare financing outcomes.

Future research should focus on: First, micro-level hospital financial behavior mechanism research, with Hashtarkhani et al.'s (37) spatial analysis of healthcare accessibility providing technical support; Second, quantitative analysis of trade-offs between financial sustainability and medical quality, with Twea et al.'s (38) hospital cost analysis in low-income countries providing reference; Third, evaluation of artificial intelligence and big data application effects in financial management; Fourth, long-term impact tracking of emerging policies. Bouzaidi and Ragbi's (39) analysis of universal health coverage in Morocco provides new perspectives for international comparative research. These studies will further enrich theoretical systems of public hospital financial sustainability and provide more precise empirical support for policy optimization.

## 6 Conclusion

Chinese public hospital financial sustainability exhibited “fluctuating rise with intensifying regional differentiation” spatio-temporal evolution characteristics during 2010–2022. Temporally, it experienced three stages: steady improvement period (2010–2015), rapid growth period (2016–2019), and stable development period (2020–2022). σ convergence testing shows *V*-shaped evolution trajectories, with standard deviation decreasing from 0.186 in 2010 to 0.142 in 2016, then rising to 0.167. β convergence analysis confirmed annual convergence speed of 1.8%, with policy shock effects having 2–3 years lag periods. Spatially, it exhibits significant “high east-low west, strong south-weak north” distribution patterns, with global Moran's *I* index of 0.347. Club convergence analysis identified high, medium, and low-level convergence clubs including 9, 10, and 12 provinces, respectively.

Driving mechanism analysis indicates that government fiscal investment, medical insurance system improvement, and regional economic development are key factors, with government fiscal expenditure total effect reaching 0.401, including indirect effect 0.089 reflecting significant spatial spillover. Spatial diffusion mainly achieves through three mechanisms: policy learning, talent mobility, and technological diffusion, with adjacent provincial policy adoption correlation coefficient reaching 0.367.

This study enriches public health economics theory by providing a novel spatio-temporal analytical framework for understanding financial sustainability evolution patterns in developing country public hospitals, particularly advancing spatial econometric applications in healthcare research. The policy implications require comprehensive consideration of the relationships between efficiency and equity, central and local governance, and short-term adjustments vs. long-term sustainability, leading to the establishment of adaptive financial guarantee mechanisms. This study provides a replicable analytical framework for understanding healthcare financing dynamics in large developing countries with significant regional disparities.

## Data Availability

The original contributions presented in the study are included in the article/supplementary material, further inquiries can be directed to the corresponding author.
